# Plk1 Mediates Paxillin Phosphorylation (Ser-272), Centrosome Maturation, and Airway Smooth Muscle Layer Thickening in Allergic Asthma

**DOI:** 10.1038/s41598-019-43927-8

**Published:** 2019-05-17

**Authors:** Alyssa C. Rezey, Brennan D. Gerlach, Ruping Wang, Guoning Liao, Dale D. Tang

**Affiliations:** Department of Molecular and Cellular Physiology, Albany Medical College, 47 New Scotland Avenue, MC-8, Albany, New York USA

**Keywords:** Cell growth, Respiration

## Abstract

Allergic asthma is characterized by airway smooth muscle layer thickening, which is largely attributed to cell division that requires the formation of centrosomes. Centrosomes play a pivotal role in regulating bipolar spindle formation and cell division. Before mitosis, centrosomes undergo maturation characterized by expansion of pericentriolar material proteins, which facilitates spindle formation and mitotic efficiency of many cell types. Although polo-like kinase 1 (Plk1) has been implicated in centrosome maturation, the mechanisms by which Plk1 regulates the cellular process are incompletely elucidated. Here, we identified paxillin as a new Plk1-interacting protein in human airway smooth muscle cells. We unexpectedly found that phosphorylated paxillin (Ser-272) was localized in centrosomes of human smooth muscle cells, which regulated centrosome maturation and spindle assembly. Plk1 knockdown inhibited paxillin Ser-272 phosphorylation, centrosome maturation, and cell division. Furthermore, exposure to allergens enhanced airway smooth muscle layer and paxillin phosphorylation at this residue in mice, which was reduced by smooth muscle conditional knockout of Plk1. These findings suggest that Plk1 regulates centrosome maturation and cell division in part by modulating paxillin phosphorylation on Ser-272. Furthermore, Plk1 contributes to the pathogenesis of allergen-induced thickening of the airway smooth muscle layer by affecting paxillin phosphorylation at this position.

## Introduction

Airway smooth muscle layer thickening is one of the key pathological characteristics of allergic asthma, which worsens airway obstruction during asthma attacks^1,2^. Increases in thickness of the airway smooth muscle layer are largely attributed to enhanced cell division^1–3^, which requires the formation of centrosomes that are composed of a pair of centrioles embedded in pericentriolar material (PCM) and function as microtubule organizing centers^4^. Before mitosis, many PCM proteins are recruited to centrosomes, known as centrosome maturation, which facilitates bipolar spindle formation and mitotic efficiency of many cell types. The process is then reversed during mitotic exit^4,5^.

Although more than ten proteins are implicated in centrosome maturation, several proteins are particularly interesting. Pericentrin (PCNT) is an integral component of the centrosome that serves as a multifunctional scaffold for anchoring numerous proteins and protein complexes^4^. PCNT is widely used as a marker of centrosome^4,5^. CEP192 is an early centrosome protein that has a critical function in centriole duplication, centrosome maturation, and bipolar spindle formation^6^. γ-tubulin localized in the centrosome is important for nucleation and bipolar orientation of microtubules during maturation^4,5,7,8^. More importantly, the mitotic serine/threonine protein kinase polo-like protein kinase 1 (Plk1) is recruited to the early centrosome by CEP192 and plays a particularly important part in the maturation process^4,9,10^. Plk1 also participates in the regulation of smooth muscle functions including proliferation, migration, and contraction^11–14^. Several potential substrates of Plk1 have been identified; Plk1 interacts with and phosphorylates PCM proteins including PCNT, axin and centrosomin, which promotes centrosome maturation during mitosis^4,5,7,8^. However, Plk1 may also regulate other proteins during centrosome maturation.

Paxillin is an actin-associated protein that has been implicated in focal adhesion formation, cell adhesion, migration, survival, and smooth muscle contraction^2,15–18^. Rodent paxillin undergoes phosphorylation at Ser-273 (equivalent to human paxillin Ser-272, NCBI Accession number, NP_002850.2) during migration of CHO-K1 cells, which regulates adhesion dynamics^19^. Paxillin also gets phosphorylated at Tyr-31 and Tyr-118 during adhesion, migration, and contraction^2,15,17,20^. The role of paxillin in centrosome maturation and bipolar spindle assembly has not been previously investigated.

Here, we identify paxillin as one of the major proteins complexing with Plk1 in human airway smooth muscle (HASM) cells. Phosphorylated paxillin (Ser-272) mediated by Plk1 is localized in centrosomes, which regulates centrosome maturation, spindle formation, and cell division. More importantly, Plk1 contributes to the progression of airway smooth muscle layer thickening in mice exposed to allergens.

## Results

### Paxillin is one of the major proteins complexing with Plk1 in smooth muscle cells

**Figure 1:**
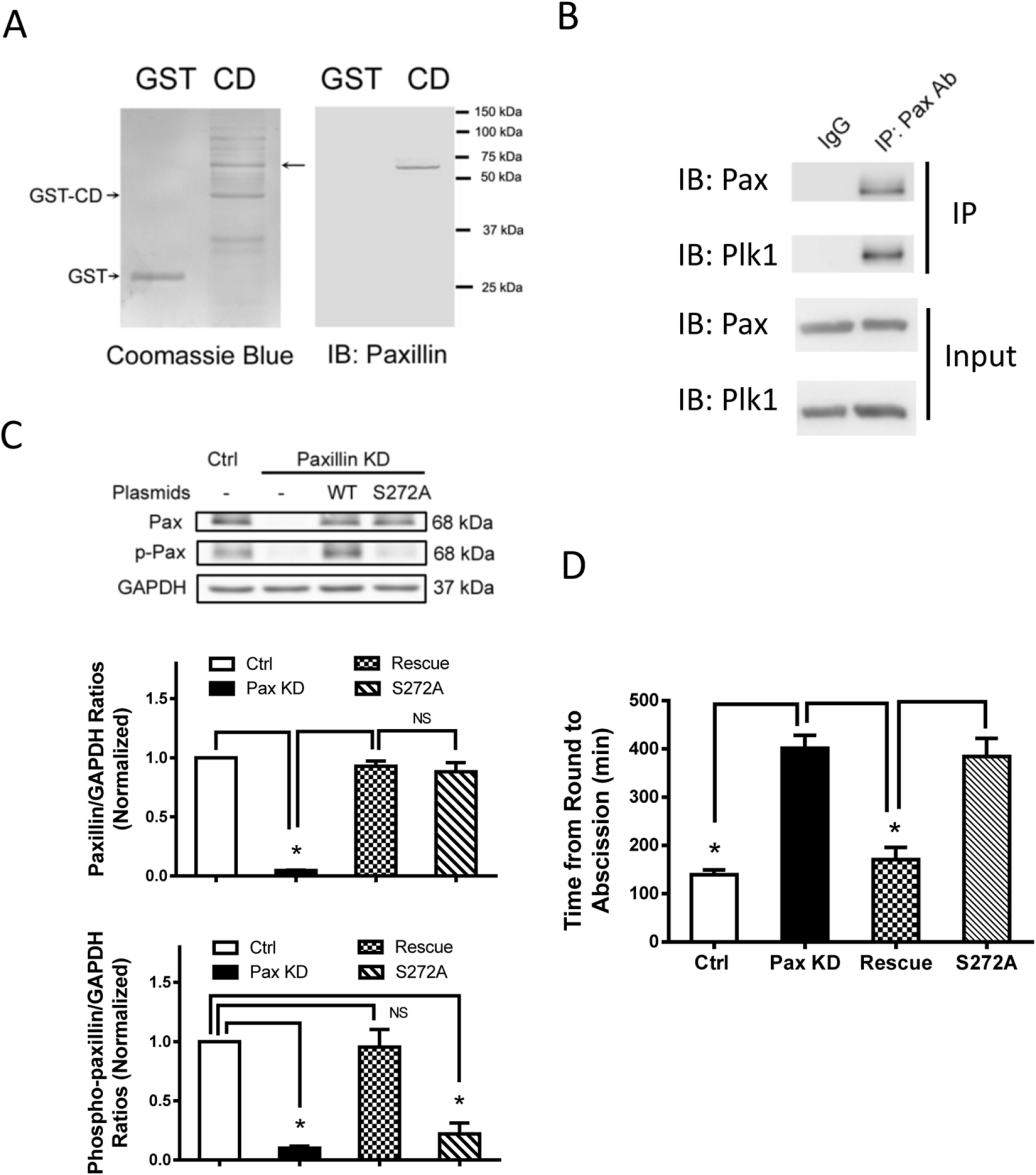
Paxillin and its phosphorylation at Ser-272 are important for smooth muscle cell division. (**A**) Plk1-interacting proteins were precipitated with the GST-tagged Plk1 catalytic domain (GST-CD), and separated by SDS-PAGE. In separate experiment, precipitated proteins were analyzed by immunoblot analysis. Paxillin is one of the major Plk1-interacting proteins in HASM cells. The images are representative of three experiments. (**B**) Extracts of HASM cells were immunoprecipitated with paxillin antibody and blotted with paxillin antibody followed by membrane stripping. The membranes were reblotted with Plk1 antibody. Blots for input are also shown. Blots are representative of three experiments. (**C**) Representative immunoblots illustrating paxillin (Pax) knockdown (KD) and recombinant protein expression in cells. Blots of HASM cells transfected with control (Ctrl) siRNA or Pax siRNA with or without the DNA constructs for 2 days were probed with antibodies against Pax, phospho-paxillin (Ser-272) and GAPDH. Ratios of Pax/p-Pax over GAPDH in Pax KD, rescue, and S272A cells are normalized to ratios obtained from cells treated with Ctrl siRNA. WT, wild type Pax. Data are mean ± SE (n = 4). *P < 0.05. NS, not significant. (**D**) Cells were plated on 6-well dishes and cultured for 5 hours to allow for cell attachment. Cell division was monitored live using a time-lapse microscope for additional 24 hours. The time from round shape to cytokinetic abscission is increased in paxillin KD cells and cells expressing S272A paxillin, but restored in rescue cells. Data are mean ± SE, n = 32–39 cells. *P < 0.05. One-way analysis of variance was used for statistical test.

To identify proteins complexing with Plk1, we used an unbiased affinity precipitation assay. Briefly, extracts of HASM cells were reacted with the GST-Plk1 catalytic domain followed by the addition of glutathione beads. Plk1-associated proteins were precipitated and separated by SDS-PAGE. Coomassie blue staining of the gels showed that a protein with 68 kDa molecular weight was one of the major Plk1-associated proteins (Fig. 1A). This protein was later identified as paxillin by immunoblot analysis (Fig. 1A). To verify this, we immunoprecipitated paxillin from HASM cell extracts using anti-paxillin followed by immunoblot analysis. Plk1 was found in paxillin immunoprecipitates from smooth muscle cells (Fig. 1B).

### Paxillin regulates division of smooth muscle cells

Because paxillin associates with Plk1, which is known to regulate mitosis, we questioned whether paxillin has a role in cell division. HASM cells were treated with control or paxillin siRNA for 2 days. Treatment with paxillin siRNA reduced paxillin protein expression significantly (Fig. 1C). To assess whether paxillin is involved in division, control and paxillin KD cells were plated on cell dishes and cultured for 5 hours to allow for cell attachment. Cell behavior was monitored live using a time-lapse microscope for additional 24 hours. Smooth muscle cells became a round shape (indication of mitotic entry) followed by furrow ingression and cytokinetic abscission during division. Quantification of live cell images showed that cells treated with control siRNA required approximately 145 min to cytokinetic abscission from a round shape (Figs 1D and S1). However, paxillin KD cells required 400 min to complete abscission from round shapes (Figs 1D and S1). Furthermore, we performed rescue experiment, in which cells were co-transfected with paxillin siRNA plus RNAi-resistant paxillin construct for 2 days followed by time-lapse microscopy. Paxillin rescue recovered the timing for cell division (Fig. 1C,D).

### Paxillin regulates bipolar spindle formation and centrosome maturation

**Figure 2:**
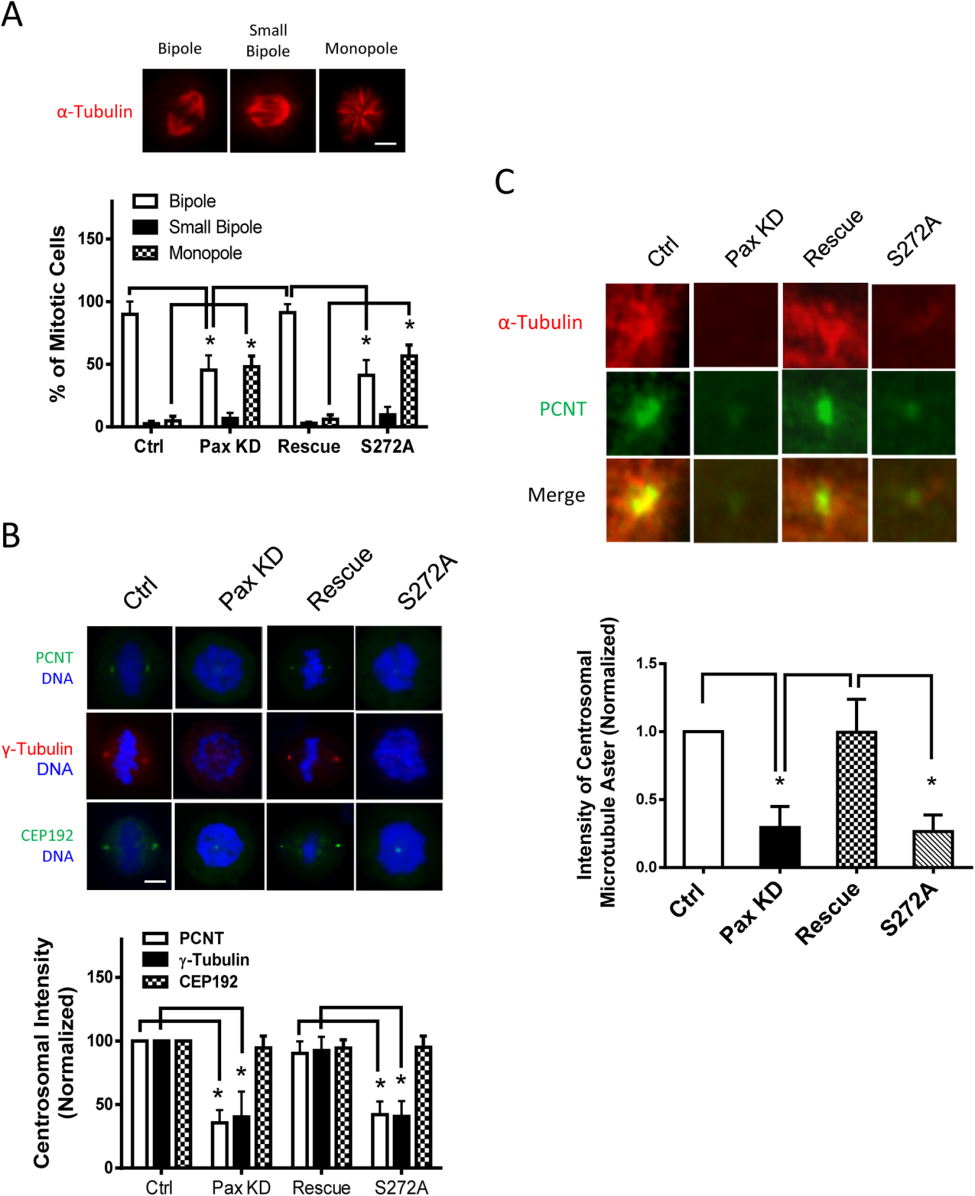
Bipolar spindle formation, centrosome maturation, and microtubule growth are regulated by paxillin and its phosphorylation at Ser-272. (**A**) Cells were immunostained for α-tubulin to visualize mitotic spindle. The phenotypes of HASM cells transfected with control or paxillin siRNA with or without the plasmids are classified as bipole, small bipole, and monopole. Data represent mean ± SE (n = 45–49 cells). *P < 0.05. Scale bar, 5 μm. (**B**) HASM cells under various treatments were synchronized and released (washed out) using the experimental procedures described in “Methods.” The cells were immunostained with indicated antibodies. Cells were also stained with DAPI to detect DNA. Scale bar, 5 µm. Centrosomal intensities of each labeled protein are normalized to cells treated with control siRNA. Data represent mean ± SE (n = 45–53 cells). *P < 0.05. (**C**) Cells under various treatments were incubated with nocodazole for 3 h and placed in ice for 1 h to depolymerize microtubules. Cells were then transferred to nocodazole-free medium at 37 °C 30 min for regrowth followed by immunostaing for indicated proteins. Intensity of centrosomal microtubule aster is normalized to cells treated with control siRNA. Data are mean ± SE (n = 48–52). *P < 0.05. One-way analysis of variance was used for statistical test.

Since bipolar spindle formation is critical for cell division, we questioned whether paxillin is involved in spindle formation. Compared to control cells, mitotic cells with monopole or small bipoles were increased in paxillin KD cells (Fig. 2A). Moreover, dividing cells with bipoles were decreased in paxillin KD cells (Fig. 2A). However, paxillin rescue in the paxillin KD cells restored normal spindle formation (Fig. 2A). These results suggest that paxillin KD induces defects in spindle formation.

Because centrosome maturation is important for bipolar spindle assembly of somatic cells^4,5^, we evaluated the effects of paxillin KD on the cellular localization of PCNT (a major PCM protein for centrosome maturation) and γ-tubulin which has a role in nucleation and polar orientation of microtubules during maturation^4,5,7,8^. Fluorescent intensity of PCNT and γ-tubulin in the centrosome was diminished in paxillin KD cells, which was restored in paxillin rescue cells (Fig. 2B). However, paxillin KD and rescue did not affect the centrosomal intensity of CEP192 (Fig. 2B), another PCM protein^4^. The results also indicate that paxillin KD does not have non-specific impact on centrosome structure. Furthermore, because centrosome maturation has been implicated in microtubule formation, we assessed the role of paxillin in microtubule regrowth. The intensity of α-tubulin adjacent to PCNT was lower in paxillin KD cells, which was recovered in paxillin rescue cells (Fig. 2C). These results suggest that paxillin modulates centrosome maturation and microtubule regrowth.

### Phosphorylated paxillin at Ser-272 (pS272-paxillin) is localized in the centrosome during mitosis

**Figure 3:**
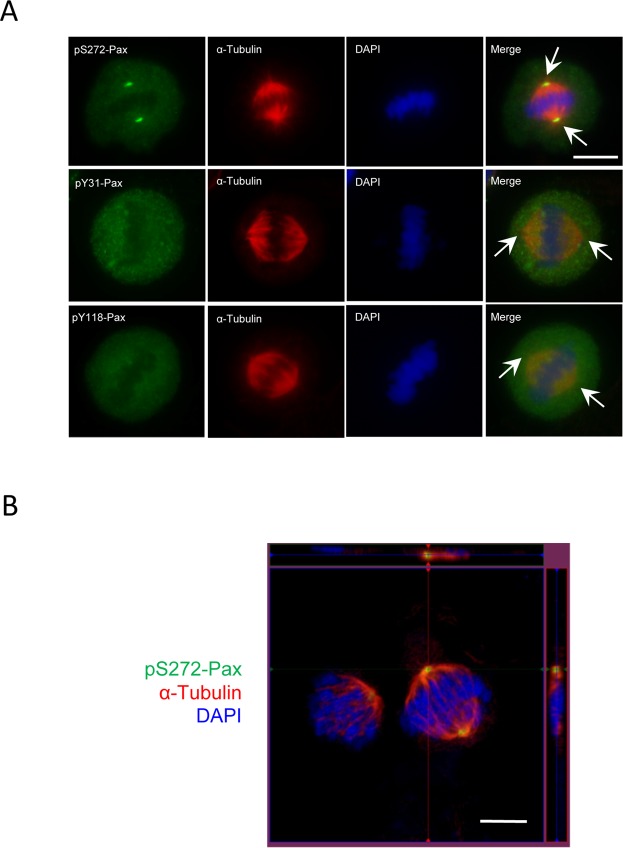
Phosphorylated paxillin (Ser-272) is localized in spindle poles during mitosis. (**A**) Mitotic cells were plated on coverslips and were stained with antibodies against phospho-paxillin (pS272-paxillin, pY31-paxillin or pY118-paxillin) and α-tublin. Nuclei were stained using DAPI. pS272-paxillin, but not pY31-paxillin and pY118-paxillin, is localized in the spindle poles. Scale bar: 10 μm. Arrows point to spindle poles. (**B**) Z stack images of cells showing connection of microtubule spindle with pS272-paxillin in the poles. Scale bar, 5 μm.

Because rodent paxillin phosphorylation at Ser-273 (equivalent to human paxillin Ser-272) has been implicated in regulating adhesion dynamics^19^, we hypothesized that paxillin phosphorylation at Ser-272 may have a role in cell division. To test this, we evaluated the spatial distribution of pS272-paxillin during mitosis using a wide field fluorescent microscope. We unexpectedly found that pS272-paxillin was localized in spindle poles in metaphase (Fig. 3A). Because paxillin also gets phosphorylated at Tyr-31 and Tyr-118 during adhesion, migration, and contraction^2,15,17,20^, we determined the spatial distribution of pY31-paxillin (paxillin phosphorylation at Tyr-31) and pY118-paxillin (paxillin phosphorylation at Tyr-118) in mitotic cells. pY31-paxillin and pY118-paxillin were not positioned in the spindle poles (Fig. 3A).

To further investigate this, we generated Z-stack images of mitotic cells using a Zeiss LSM880 confocal microscope with Airyscan. pS272-paxillin was localized in the spindle poles of cells in metaphase. Microtubule spindle fibers extended from the pole region where pS272-paxillin resided (Fig. 3B). These novel findings suggest that pS272-paxillin, but not pY31-paxillin and pY118-paxillin, is localized in the centrosome during mitosis of human smooth muscle cells.

### Paxillin phosphorylation at Ser-272 regulates centrosome maturation, spindle formation, and cell division

Next, we determined whether paxillin phosphorylation at Ser-272 regulates centrosome maturation, paxillin KD cells were transfected with constructs for WT paxillin or the nonphosphorylated paxillin mutant S272A. Immunoblot analysis verified expression of the recombinant proteins (Fig. 1C). Moreover, the level of paxillin Ser-272 phosphorylation was lower in cells expressing S272A mutant than in cells expressing WT paxillin (Fig. 1C). Immunofluorescent microscopy was used to evaluate spatial distribution of PCNT, γ-tubulin, and CEP192. Immunofluorescent analysis showed that WT paxillin was present in the centrosome. But, S272A paxillin mutant was not detected in the centrosome (Fig. S2). Moreover, PCNT, γ-tubulin, and CEP192 were found in centrosomes of cells re-expressing WT paxillin (Fig. 2B). However, expression of S272A paxillin in the KD cells reduced the centrosomal positioning of PCNT and γ-tubulin without affecting CEP192 (Fig. 2B). Furthermore, expression of S272A paxillin also impaired bipolar spindle assembly (Fig. 2A), microtubule regrowth (Fig. 2C), and division (Fig. 1D). These results suggest that paxillin phosphorylation at Ser-272 participates in the regulation of centrosome maturation, bipolar spindle formation, microtubule growth, and division.

### Plk1 regulates centrosomal pS272-paxillin, PCNT, and γ-tubulin

**Figure 4:**
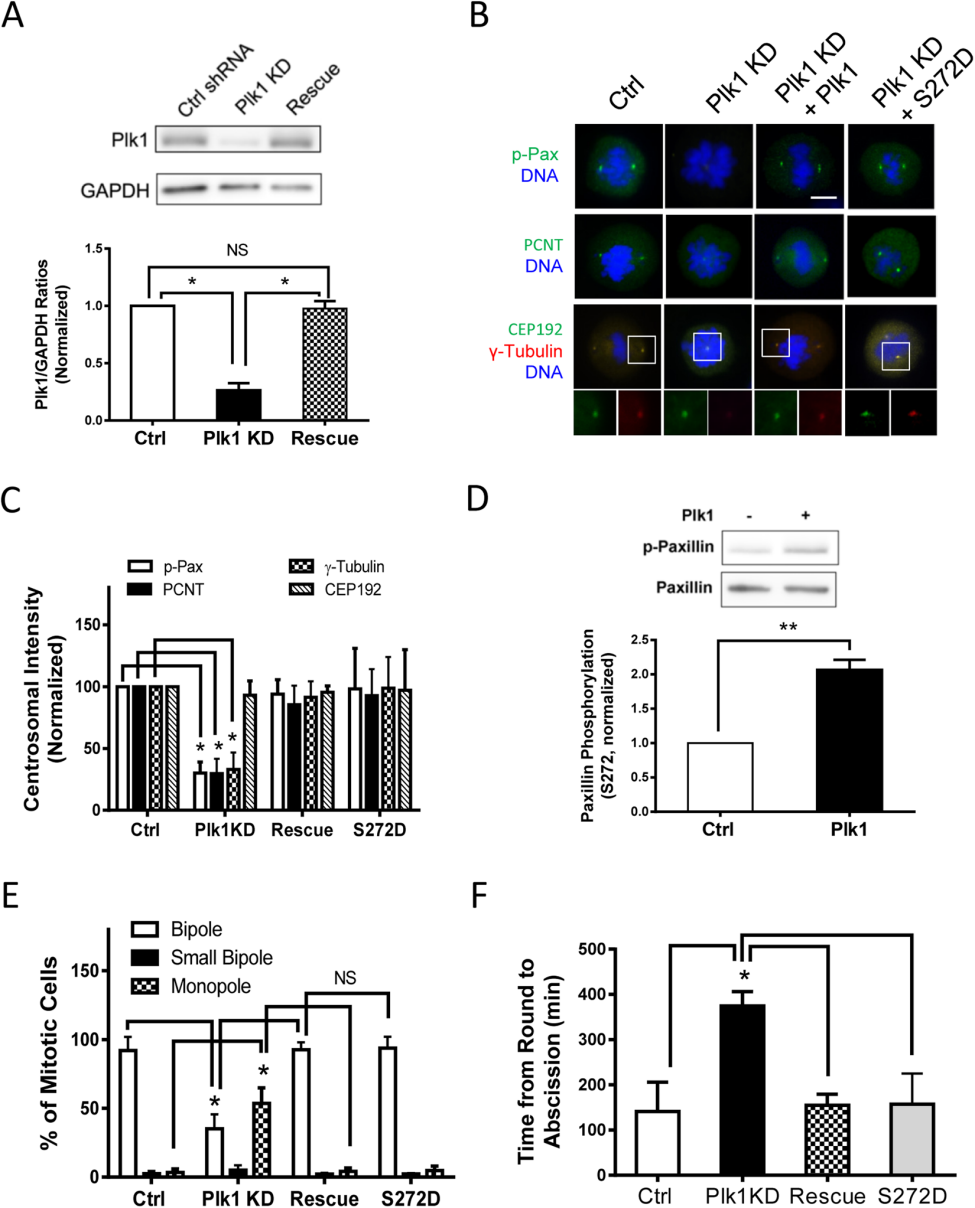
Plk1 mediates centrosomal pS272-paxillin, centrosome formation, spindle assembly, and cell division. (**A**) Blots of cells expressing control (Ctrl) shRNA or Plk1 shRNA with or without rescue construct for 2 days were probed with antibodies against Plk1 and GAPDH. Ratios of Plk1/GAPDH protein in paxillin KD and rescue cells are normalized to ratios obtained from cells expressing control shRNA. Data are mean ± SE (n = 5). *P < 0.05. NS, not significant. (**B**) Mitotic cells were immunostained with indicated antibodies and DAPI. Scale bar, 5 µm. Ctrl, control shRNA; KD, knockdown. Centrosome formation is affected by Plk1 KD and rescue. Expression of S272D paxillin restores centrosome formation in Plk1 KD cells. (**C**) Centrosomal intensities of each labeled protein are normalized to cells treated with control shRNA. Data represent mean ± SE (n = 42–46 cells). *P < 0.05. (**D**) Purified active Plk1 (40 ng) and 1 μg purified paxillin were placed in the kinase buffer. Site-specific paxillin phosphorylation (Ser-272) was determined by immunoblot analysis 30 min after the initiation of the reaction (**P < 0.01, n = 3). (**E**) Plk1 KD and rescue affect bipolar spindle formation. Expression of S272D paxillin recovers the spindle formation in Plk1 KD cells. Data are mean ± SE (n = 48–53 cells). *P < 0.05. Scale bar, 5 μm. (**F**) Plk1 KD and rescue affect the time interval between round shape and cytokinetic abscission. Expression of S272D paxillin restores the time interval between round shape and abscission in Plk1 KD cells. Data are mean ± SE, n = 37–43 cells. *P < 0.05. One-way analysis of variance was used for Fig. 4,A,C,E, and F. Students t-test was used for Fig. 4D.

Next, we questioned whether Plk1 mediates paxillin phosphorylation at Ser-272 in centrosomes. Stable Plk1 KD HASM cells were generated using shRNAs as previously described^13^ (Also see methods). To rescue Plk1, Plk1 KD cells were transfected with RNAi-resistant Plk1 construct for 2 days. Immunoblot analysis showed effective Plk1 KD and rescue in smooth muscle cells (Fig. 4A). pS272-paxillin was reduced in the centrosome of Plk1 KD cells as compared to control cells (Fig. 4B). Moreover, the intensity of PCNT and γ-tubulin in the centrosome was reduced in Plk1 KD cells (Fig. 4B). Plk1 rescue restored the levels of pS272-paxillin, PCNT, and γ-tubulin in the centrosome (Fig. 4B,C). However, Plk1 KD and rescue did not affect the centrosomal level of CEP192 (Fig. 4B,C). Furthermore, Plk1 directly catalyzed paxillin phosphorylation at this residue as evidenced by the *in vitro* kinase assay (Fig. 4D). We noticed that phospho-paxillin band was slightly detected in the sample without purified Plk1 (Fig. 4D), which is similar to the results by others who used the phospho-antibody^19^. This may be due to lower affinity of the phospho-paxillin antibody to pan paxillin or non-specific reaction of the secondary antibody.

To further assess whether Plk1 regulates centrosome formation by affecting paxillin Ser-272 phosphorylation, we expressed the phosphorylation-mimicking mutant S272D paxillin in Plk1 KD cells and evaluated spatial distribution of centrosomal proteins. Expression of S272D paxillin in Plk1 KD cells restored centrosomal localization of pS272-paxillin, PCNT, and γ-tubulin (Fig. 4B). The results suggest that Plk1 regulates centrosomal maturation by controlling paxillin Ser-272 phosphorylation during mitosis of smooth muscle cells.

### Plk1 controls spindle formation and cell division

Plk1 KD resulted in an increase in mitotic cells with monopole and small bipole, and a decrease in cells with bipole. However, Plk1 rescue in the KD cells recovered normal spindle formation (Fig. 4E). In addition, compared to control cells, Plk1 KD cells required more time to complete cytokinetic separation. Rescue of Plk1 in KD cells reduced the time required for cytokinetic abscission (Fig. 4F). Furthermore, expression of S272D paxillin recovered spindle formation and cell division in Plk1 KD cells (Fig. 4E,F). These findings indicate that Plk1 coordinates centrosome maturation, spindle formation, and division by regulating paxillin phosphorylation at this residue.

### Allergen-induced airway smooth muscle layer thickening is mediated by Plk1

**Figure 5:**
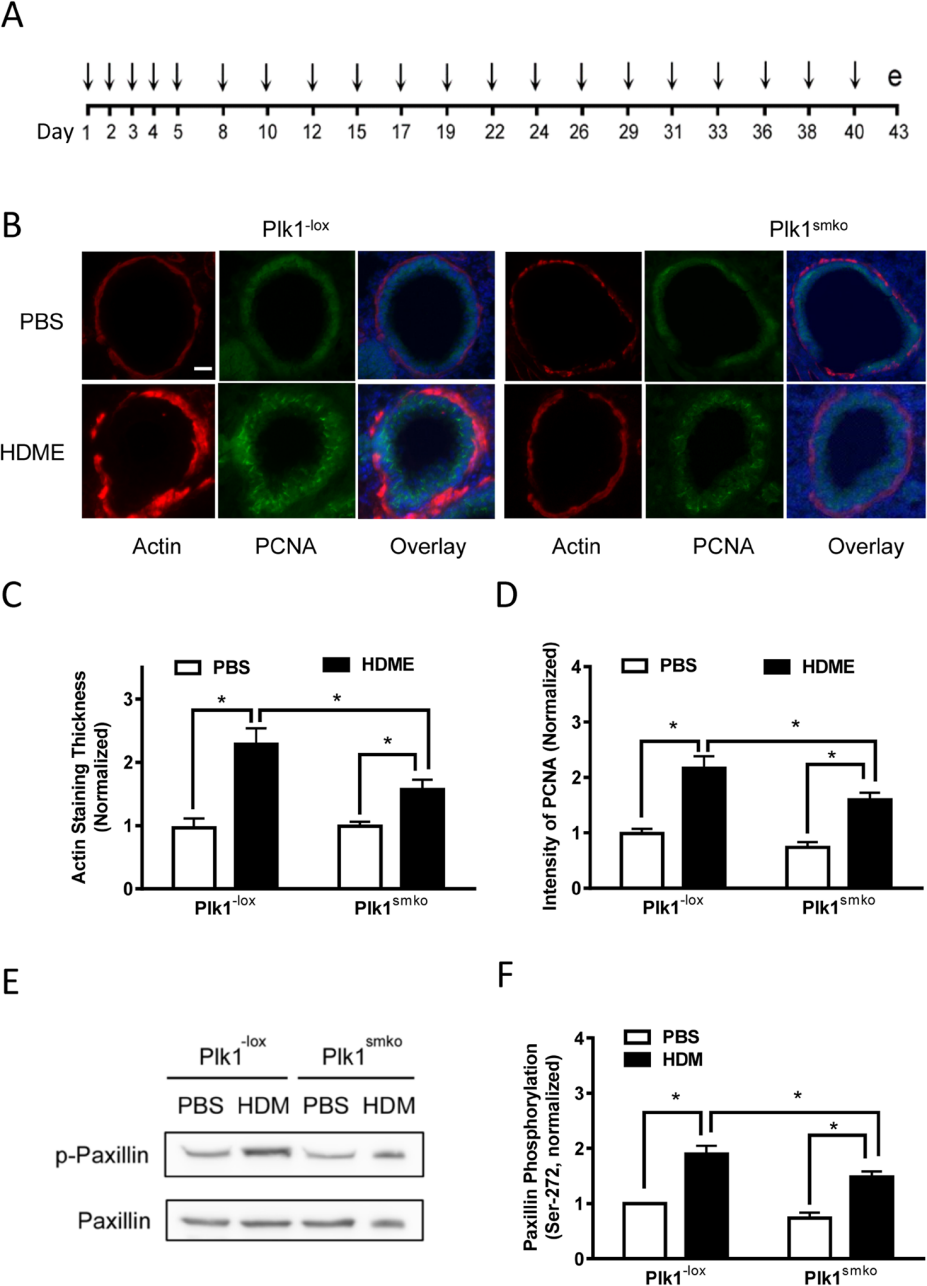
Plk1 mediates airway smooth muscle layer thickening in HDME-treated mice. (**A**) Protocol for the house dust mite extract (HDME)-treated mice. e, experiment. (**B**) Representative images illustrating the effects of Plk1 knockout on α-actin and PCNA staining. Plk1^−lox^ and Plk1^smko^ mice were exposed to HDME or PBS for six weeks. Lung sections of the mice were immunostained for α-actin (red) and PCNA (green). The sections were also counterstained with use of DAPI to visualize the nucleus (blue). Scar bar, 25 µm. (**C**) Thickness of α-actin staining in HDME-exposed Plk1^−lox^ mice is greater than that of PBS-treated Plk1^−lox^ mice. However, thickness of actin staining in Plk1^smko^ mice treated with HDME was lower compared to Plk1^−lox^ mice exposed to HDME (n = 10–11, *P < 0.05). (**D**) Fluorescent intensity of PCNA in HDME-exposed Plk1^−lox^ mice is greater than that of PBS-treated Plk1^−lox^ mice. However, intensity of PCNA in the airways in Plk1^smko^ mice treated with HDME was lower than that in Plk1^−lox^ mice exposed to HDME (n = 10–11, *P < 0.05). (**E**) Representative blots showing the effects of Plk1 knockout on paxillin Ser-272 phosphorylation in tracheal extracts of animals treated with PBS or HDME. (**F**) HDME exposure increases paxillin Ser-272 phosphorylation in airway tissues of Plk1^−lox^ mice, which is reduced in Plk1^smko^ mice (*P < 0.05, n = 6–7). Two-way analysis of variance was used for statistical test.

Because Plk1 regulates cell division, we questioned whether Plk1 plays a role in airway smooth muscle growth, a key feature of allergic asthma^1–3,21^. Plk1 smooth muscle conditional knockout (Plk1^smko^) mice were generated by crossing Plk1^−lox^ mice with Myh11-cre mice as previously described^13^. Plk1^smko^ and Plk1^−lox^ mice were exposed to house dust mite extracts (HDME) or PBS (control) intranasally for 6 weeks (Fig. 5A). HDM is a major perennial allergen source and a significant cause of allergic asthma^22^. Smooth muscle conditional knockout of Plk1 was verified in airway tissues of Plk1^smko^ mice^13^ (Fig. S3A). Moreover, the HDME-exposed mice showed asthma-like symptoms such as airway hyperresponsiveness^13^ (Fig. S3B).

Exposure to HDME increased the thickness of α-smooth muscle actin staining (indication of the smooth muscle layer) in the airways of Plk1^−lox^ mice, which was reduced in Plk1^smko^ mice (Fig. 5B,C). The fluorescent intensity of α-smooth muscle actin staining was higher in Plk1^−lox^ mice treated with HDME as compared to naïve Plk1^−lox^ mice, suggesting higher α-smooth muscle actin expression in remodeled airways in the animal model^23,24^. Moreover, the fluorescent intensity of proliferating cell nuclear antigen (PCNA)^23,25^ was greater in the airways of Plk1^−lox^ mice treated with HDME, which was reduced by Plk1 knockout (Fig. 5B,D). Furthermore, exposure to the allergen increased paxillin phosphorylation at Ser-272 in tracheal extracts of Plk1^−lox^ mice, which was reduced in Plk1^smko^ mice (Fig. 5E,F).

### HDME-induced airway inflammation was not affected by smooth muscle conditional knockout of Plk1

**Figure 6:**
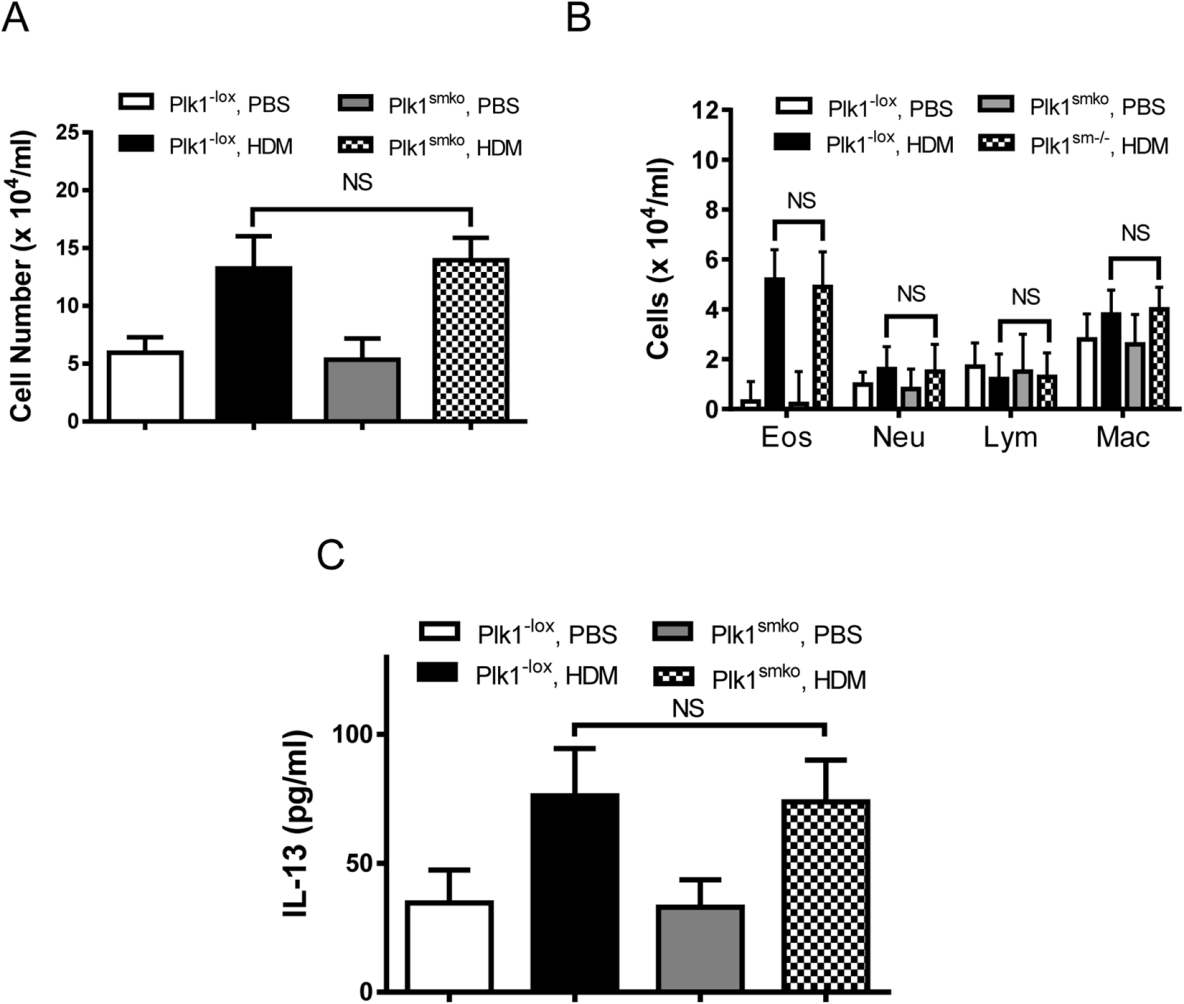
Smooth muscle conditional knockout of Plk1 does not affect increases of inflammatory cells and IL-13 production in HDME-exposed mice. Total (**A**) and differential (**B**) cell numbers in bronchoalveolar lavage fluid (BALF) of Plk1^smko^ mice are similar to those of Plk1^−lox^ mice when treated with PBS or HDM allergen (n = 9–10). (**C**) The levels of IL-13 in BALF of Plk1^smko^ mice are similar to those of Plk1^−lox^ mice (n = 9–10). NS, not significant. Two-way analysis of variance was used for statistical test.

In asthmatic patients and animal models, inflammatory cells infiltrate into the lungs and IL-13 levels are enhanced in the bronchoalveolar space, which are key characteristic of allergic airway inflammation^1,26^. Because both airway smooth muscle cell growth and airway inflammation occur in allergic asthma^1,23^, this raises the possibility that Plk1-mediated airway smooth muscle thickening may result from allergic airway inflammation. Thus, we evaluated whether the smooth muscle-specific depletion of Plk1 affects recruitment of inflammatory cells by assessing total and differential cell counts of bronchoalveolar lavage fluid (BALF) in lungs of naïve and allergen-treated Plk1^−lox^ mice and Plk1^smko^ mice. Exposure to HDME increased the numbers of total and differential cells in the lungs of Plk1^−lox^ mice. However, the allergen-induced increase in cell numbers in the lungs in Plk1^smko^ mice was similar to that in Plk1^−lox^ mice (Fig. 6A,B).

To determine the role of Plk1 in smooth muscle in the production of IL-13, a key cytokine in asthma pathogenesis^26^, we evaluated the level of IL-13 in the BALF in lungs of naïve and HDME-treated Plk1^−lox^ and Plk1^smko^ mice. Exposure to HDME increased the level of IL-13 in the BALF of Plk1^−lox^ mice. Interestingly, the allergen-induced increase of IL-13 in the lungs of Plk1^smko^ mice was similar to those in Plk1^−lox^ mice (Fig. 6C). These results suggest that the expression of Plk1 in smooth muscle does not affect allergic airway inflammation.

## Discussion

Plk1 has been implicated in various cell functions including centrosome maturation, spindle assembly, proliferation, and smooth muscle contraction^2,9,11,13,14,27,28^. In this report, we identified paxillin as a new Plk1-interacting protein in smooth muscle cells, an important cell type for the regulation of respiratory and circulatory systems^11,17,29,30^. Furthermore, we found that paxillin was necessary for centrosome maturation, mitotic spindle assembly, and cell division (Figs 1 and 2). Additionally, in non-mitotic breast cancer cells, paxillin was required for centrosome cohesion, which may be associated with cell polarity during migration^31^. Thus, these two independent investigations suggest that paxillin is involved in structural changes of centrosomes in human cells.

Because paxillin phosphorylation at Ser-272 has been implicated in protrusion dynamics that is associated with F-actin formation^19^, our original hypothesis was that pS272-paxillin localizes to the contractile ring (also mediated by F-actin formation)^32,33^, and regulates contractile ring assembly. However, current studies unexpectedly discovered that pS272-paxillin localizes to the centrosome (Fig. 3). Moreover, paxillin phosphorylation at this residue modulates the recruitment of PCNT and γ-tubulin, and bipolar spindle assembly. Because microtubules are nucleated and anchored by γ-tubulin ring complexes embedded within the centrosome’s PCM^4^, paxillin phosphorylation on Ser-272 may affect spindle formation by modulating centrosome maturation.

Although paxillin phosphorylation at Tyr-31 and Tyr-118 has been implicated in smooth muscle function^2,34^, pY31-paxillin and pY118-paxillin were not detected in the centrosome of mitotic smooth muscle cells (Fig. 3). The results suggest that paxillin phosphorylation at these two tyrosine sites may not be involved in centrosome formation of mitotic smooth muscle cells. Interestingly, WT paxillin, but not Y31/118F paxillin mutant, was localized in the centrosome of non-mitotic breast cancer cells and U2OS cell^31^. The results suggest that paxillin phosphorylation at Tyr-31 and Tyr-118 appears to modulate its localization in the centrosome of non-mitotic cells^31^. The discrepancy could be due to non-mitotic state vs. mitotic state and cancer cells vs. smooth muscle cells.

Plk1 KD reduced paxillin phosphorylation at Ser-272 in mitotic centrosomes (Fig. 4). To the best of our knowledge, this is the first evidence that Plk1 mediates paxillin phosphorylation at this position during centrosome maturation. Because Plk1 is recruited to early centrosomes^4,5^, it is likely that paxillin is recruited to the centrosome, and phosphorylated by centrosomal Plk1 during mitosis. However, we do not rule out the possibility that paxillin may get phosphorylated in the cytoplasm and then recruited to centrosomes. In addition, in migratory human prostate cancer cells, p21-activated kinase 4 (PAK4) was localized at focal adhesions, and was immunoprecipitated with paxillin and phosphorylated paxillin on Ser-272^35^. Moreover, PAK1 catalyzed murine paxillin phosphorylation at Ser-273 (similar to human paxillin at Ser-272) in an *in vitro* transcription–translation coupled kinase assay, which may increase migration, protrusion, and adhesion turnover of murine cells^19^. We do not exclude the possibility that PAK may mediate paxillin phosphorylation on Ser-272 during mitosis.

The expression of S272A paxillin and Plk1 KD hindered the positioning of PCNT and γ-tubulin in the centrosome without affecting centrosomal localization of CEP192 (Figs 2 and 4). Previous studies by others suggest that CEP192 is an early centrosome protein that recruits other proteins including Plk1 to the centrosomes and activate them in Hela cells^10^. Thus, it is not surprising that the expression of S272A paxillin and Plk1 KD did not affect CEP192 recruitment. Furthermore, Plk1 mediates paxillin phosphorylation at this position in mitotic cells (Fig. 4B), and expression of phosphorylation-mimicking paxillin mutant restores centrosome maturation, spindle assembly, and division (Fig. 4B,C,E,F). It is likely that during early stage of centrosome assembly, CEP192 recruits Plk1, which catalyzes paxillin Ser272 phosphorylation, altering centrosomal microenvironment and promoting the recruitment of PCNT, and γ-tubulin and bipolar spindle formation.

In this report, exposure to the allergen induced airway smooth muscle layer thickening and paxillin phosphorylation at Ser-272 in mice. More importantly, smooth muscle conditional knockout of Plk1 reduced the allergen-induced thickening of the airway smooth muscle layer and paxillin phosphorylation at this position. Considering the role Plk1 in paxillin phosphorylation on Ser-272 and division in the cell model, it is likely that Plk1 contributes to airway smooth muscle layer thickening in allergic asthma in part by affecting paxillin phosphorylation and smooth muscle cell division. Because Plk1 also has a role in regulating the mitogen-activated protein kinase (MAPK) pathway and migration in smooth muscle cells^12,14^, the allergen-induced airway smooth muscle layer thickening may also be mediated by Plk1-mediated MAPK activation and smooth muscle cell migration. Airway smooth muscle cell migration has been linked to remodeling of human asthmatic airways^17,36,37^. Furthermore, our recent studies indicate an important role for Plk1 in airway hyperresponsiveness in animals exposed to allergens^13^. Thus, these investigations suggest that Plk1 is involved in the progress of airway smooth muscle layer thickening and hyperresponsiveness in allergic asthma.

Airway inflammation characterized by increases in inflammatory cells and IL-13 is another feature of allergic asthma^26,38^. There is evidence to suggest that allergen exposure enhances both airway smooth muscle growth and airway inflammation^1,23^. This raises the possibility that Plk1-mediated airway smooth muscle thickening may stem from allergic airway inflammation. However, conditional knockout of Plk1 did not affect airway inflammation in the animal model (Fig. 6). The results support the concept that Plk1-mediated airway smooth muscle thickening in allergic asthma results from the effects of Plk1 on cell division, but not from allergic airway inflammation.

**Figure 7:**
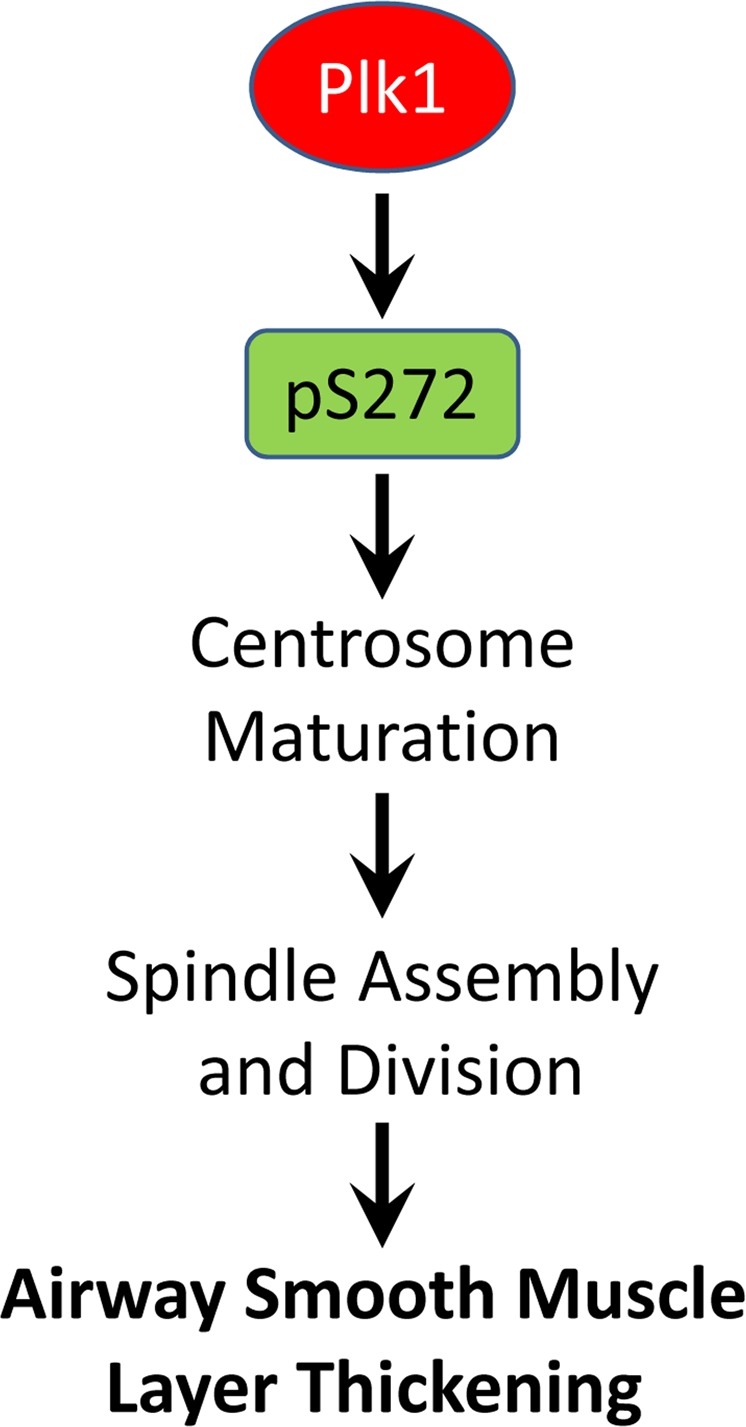
Proposed model. During mitosis, Plk1 mediates paxillin phosphorylation at Ser-272, which facilitates centrosome maturation, bipolar spindle assembly, and cell division. Plk1 also contributes to the development of airway smooth muscle layer thickening in part by modulating paxillin phosphorylation-associated process.

Taken together, our results identify paxillin as a new downstream target of Plk1 and unveil an unexpected role of paxillin in centrosome maturation and cell division. Plk1 coordinates paxillin phosphorylation at Ser-272, centrosome maturation, spindle assembly, and cell division. Moreover, Plk1 may contribute to the pathogenesis of allergen-induced airway smooth muscle layer thickening in part by modulating paxillin phosphorylation on Ser-272 (Fig. 7).

## Methods

### Cell Culture

HASM cells were prepared from human bronchi and adjacent tracheas obtained from the International Institute for Advanced Medicine^21,39–41^. Human tissues were non-transplantable, and informed consent were obtained for research. This study was approved by the Albany Medical College Committee on Research Involving Human Subjects. All methods were performed in accordance with the relevant guidelines and regulations. Cells were cultured at 37 °C in the presence of 5% CO_2_ in Ham’s F12 medium supplemented with 10% (v/v) fetal bovine serum (FBS) and antibiotics (100 units/ml penicillin, 100 µg/ml streptomycin). For Plk1 KD, lentiviruses encoding Plk1 shRNA (sc-36277-V) or control shRNA (sc-108080) were purchased from Santa Cruz Biotechnology. HASM cells were infected with control shRNA lentivirus or Plk1 shRNA lentivirus for 12 hrs. They were then cultured for 3–4 days. Positive clones expressing shRNAs were selected by puromycin. Immunoblot analysis was used to determine the expression levels of Plk1 in these cells. Plk1 KD cells and cells expressing control shRNA were stable at least five passages after initial infection^13,14^.

### Antibody and siRNAs

Antibodies used were anti-Plk1 (EMD Millipore, #05-844), anti-GAPDH (Ambion, #AM4300), anti-p-paxillin (S272) (Sigma, #SAB4301321), anti-paxillin (BD Biosciences, #610052), anti-α-tubulin (Santa Cruz Biotech., SC-32293), anti-pericentrin (EMD Millipore, #ABT59), anti-γ-tubulin (Santa Cruz Biotehc., SC-17787), anti-CEP192 (Proteintech, #18832-1-AP), anti-p-paxillin (Y31) (ThermoFisher, #44-720G), anti-paxillin (Y118) (ThermoFisher, #44-722G) and anti-PCNA (ThermoFisher, #RB-9055-P). Paxillin siRNA (SC-37007) and control siRNA (SC-29439) were purchased from Santa Cruz Biotechnology.

### Affinity Precipitation and Co-immunoprecipitation Analysis

GST-Plk1 catalytic domain was generated by using Quick change II XL site-directed mutagenesis kit (Agilent Technologies). The sequence of forward primer was 5′-GGA TCC CCG AAT TCC ATG AGT GCT GCA GTG ACT GC-3′. The sequence of reverse primer was 5′-TGC GGC CGC TCG AGA AAG AAC TCG TCA TTA AGC AGC-3′. GST tagged proteins were generated using the pGEX-4T system and purified using the Bulk GST Purification Module (Amersham). Proteins were reacted with cell extracts for overnight at 4 °C, and precipitated using glutathione beads, and separated by SDS-PAGE followed by coomassie blue staining, or immunoblot analysis. Co-immunoprecipitation analysis was performed using the similar protocols as previously described^39,41–43^.

### Immunoblot Analysis

Immunoblot analysis was performed according to the methods as previously described^21,39–41^. Cells or tissues were lysed in SDS sample buffer composed of 1.5% dithiothreitol, 2% SDS, 80 mM Tris-HCl (pH 6.8), 10% glycerol and 0.01% bromophenol blue. The lysates were boiled in the buffer for 5 min and separated by SDS-PAGE. Proteins were transferred to nitrocellulose membranes. The membranes were treated with blockers (bovine serum albumin or milk) for 1 h and probed with use of primary antibodies followed by horseradish peroxidase-conjugated secondary antibodies (Thermo Fisher Scientific). Proteins were visualized by enhanced chemiluminescence (Thermo Fisher Scientific) using the GE Amersham Imager 600 system. The levels of total protein or phosphoprotein were quantified by scanning densitometry of immunoblots (GE ImageQuant TL Software). The luminescent signals from all immunoblots were within the linear range^13,32,39,42,43^.

### Mutagenesis, Plasmid Purification, and Cell Transfection

Mutagenesis, plasmid purification, and cell transfection were using similar methods as previously described^14^. S272A paxillin (alanine substitution at Ser-272) and S272D paxillin was generated by using Quick change II XL site-directed mutagenesis kit (Agilent Technologies). The template plasmid pmcherry-paxillin (Addgene plasmid #50526) was a gift of Kenneth Yamada of National Institute of Dental and Craniofacial Research. For S272A mutant, sequence of the 5′-primer was 5′- GGA CGA GCT GAT GGC TGC GCT GTC GGA TTT CAA G-3′. The 3′ primer was 5′- CTT GAA ATC CGA CAG CGC AGC CAT CAG CTC GTC C- 3′. For S272D mutant, the 5′ primer was 5′-GAC CTG TCG GAT TTC AAG TTC AT-3′. The 3′ primer was 5′- AGC CAT CAG CTC GTC CAG CTC CC-3′. The primers were synthesized by ThermoFisher. The PCR product was subcloned into pcDNA3 3xFlag, and was transformed into XL10- Gold Ultracompetenet cells (Agilent Technologies). Plasmids were purified by using the Pureklink Quick Plasmid Miniprep kit (Invitrogen). DNA sequencing was performed by Genewiz. For paxillin KD experiments, cells were transfected with control or paxillin siRNA using the Lipofectamine 2000 transfection reagent according to the manual of the manufacture (Invitrogen). For rescue experiment, Plk1 or paxillin KD cells were transfected with RNAi-resistant constructs (pcDNA3-Plk1 or paxillin) using the FuGENE HD transfection reagent (Promega). After optimization with different amount of DNA constructs, 1–2 µg constructs were used to control adequate expression of recombinant proteins.

### Immunofluorescence microscopy

Cells were plated in 10-cm dishes for 24 hours. For synchronization, they were treated with 200 nM nocodazole for 20 hours. Mitotic cells were then collected by tapping the dishes gently followed by brief centrifugation. Cells were resuspended in the growth medium, and were plated onto collagen-coated coverslips and immunostained for proteins of interest. Cells were also stained with 4′, 6-diamidino-2-phenylindole (DAPI) to detect DNA. The cellular localization of labeled proteins and DNA were viewed under a high-resolution digital microscope (Leica DMI 6000) and a Zeiss LSM880 confocal microscope with Airyscan.

### *In vitro* kinase assay

Active Plk1 (40 ng, Millipore) and 1 μg paxillin were placed in 20 μl kinase buffer containing 20 mM HEPES (pH 7.5), 60 mM NaCl, 2 mM MgCl_2_, 5 mM EGTA and 100 μM ATP. Kinase reaction mix was incubated at 30 °C for 30 min, and stopped by the addition of the SDS sample buffer^14,30,44^. The samples were boiled for 5 min and separated by SDS-PAGE followed by membrane transfer. The membrane was probed with phospho-paxillin (Ser-272) antibody, stripped, and reproofed with paxillin antibody.

### Animals

All animal protocols were reviewed and approved by the Institutional Animal Care and Usage Committee (IACUC) of Albany Medical College. All experiments were strictly performed in accordance with approved protocols and regulations of IACUC. Animals were bred in the specific pathogen free housing of Animal Research Facility, Albany Medical College. Both male and female mice were randomized allocated to the experimental or control groups.

To generate smooth muscle conditional Plk1 knockout mice (Plk1^smko^ mice), Plk1^−lox^ mice (from Toronto Centre for Phenogenomics; genetic background, C57BL/6N) were crossed with B6.Cg-Tg (Myh11-cre,-EGFP) 2 Mik/J mice (from Jackson Laboratory, genetic background, C57BL/6J). Plk1^−lox^ and Plk1^smko^ mice (6–8 weeks old, male and female) were randomly exposed to HDME (50 μg of d. pteronyssinus, Greer) or PBS (control) intranasally for 5 days followed by every other day exposures weekly for 5 weeks. Immunohistochemistry of airways for these mice was performed on Day 43. The experiments were blindly performed, and results were substantiated by repetition by at least two researchers. Airway immunohistochemistry, airway resistance, and BALF cells and IL-13 was evaluated using the experimental procedures as we previously described^13,23^.

### Statistical Analysis

All statistical analysis was performed using Prism 6 software (GraphPad Software, San Diego, CA). Comparison among multiple groups was performed by one-way or two-way analysis of variance followed by Tukey’s multiple comparison test. Differences between pairs of groups were analyzed by Students t-test. Values of n refer to the number of experiments used to obtain each value. P < 0.05 was considered to be significant.

## Supplementary information

Supplementary information

## Data Availability

All data generated or analyzed during this study are included in this published article. All research materials are available upon request by qualified investigators.
