# Supplementary material for: Plk1 Mediates Paxillin Phosphorylation (Ser-272), Centrosome Maturation, and Airway Smooth Muscle Layer Thickening in Allergic Asthma
Source: Sci Rep. 2019 May 17;9:7555. doi: 10.1038/s41598-019-43927-8 (PMC6525254; doi:10.1038/s41598-019-43927-8)
Supplement: Supplementary file 1 — Supplementary information [file 41598_2019_43927_MOESM1_ESM.pdf]

Supplementary Figure

Plk1 Mediates Paxillin Phosphorylation (Ser-272), Centrosome Maturation, and Airway Smooth Muscle Layer Thickening in Allergic Asthma

Alyssa C. Rezey, Brennan D. Gerlach, Ruping Wang, Guoning Liao,

and Dale D. Tang*

Department of Molecular and Cellular Physiology

Albany Medical College

47 New Scotland Avenue, MC-8

Albany, New York, U.S.A.

*Correspondence: Dale D. Tang, Department of Molecular and Cellular Physiology, Albany Medical College, 47 New Scotland Avenue, MC-8, Albany, NY 12208
Tel: (518)-262-6416; Fax: (518)-262-8101; E-mail: tangd@mail.amc.edu

a

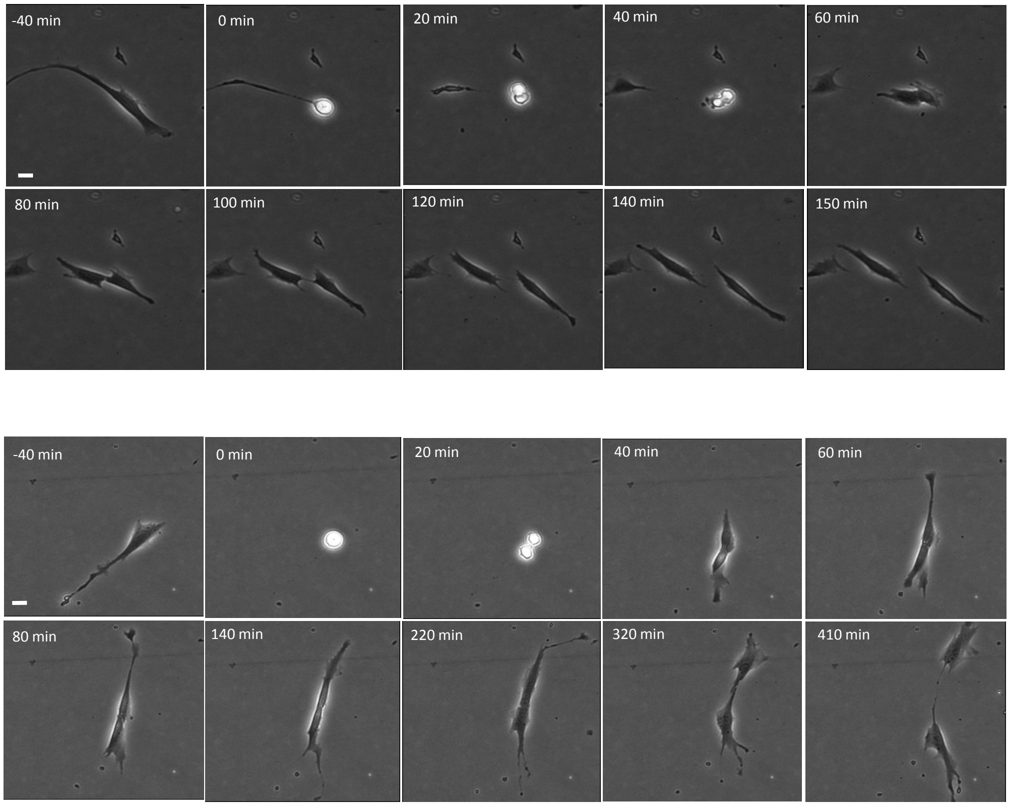

Figure S1. Paxillin KD increases time from round shape to cytokinetic abscission. Human airway smooth muscle cells treated with control (a) or paxillin (b) siRNA were plated on cell dishes and cultured for 5 hours. Cell behavior was monitored live using a time-lapse microscope (Leica DMI6000) for additional 24 hours. Paxillin knockdown cells requires longer time for division. Scar bar: 25 µm.

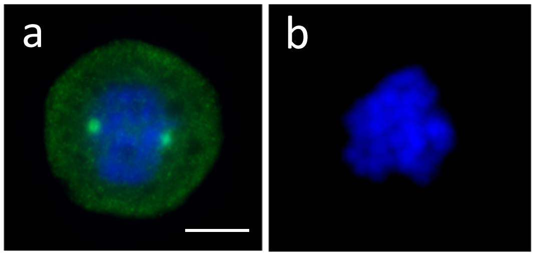

Figure S2. Wild type (a), but not S272A (b) paxillin localizes in the centrosome. HASM cells expressing wilt type or S272A paxillin were synchronized and released (washed out). The cells were immunostained with p-Pax (S272) antibody. Cells were also stained with DAPI to detect DNA. Scale bar, 5 μm. (Anti-p-Pax, anti-PCNT, and anti-CEP192 are rabbit polyclonal antibodies, we can't costain cell with these antibodies).

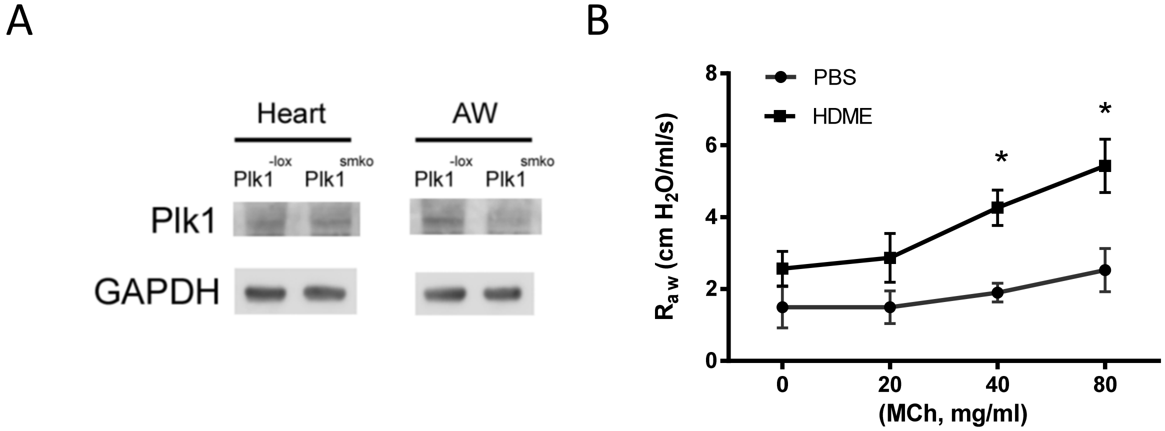

Figure S3. (A) Analysis of Plk1 smooth muscle conditional knockout of mice by immunoblotting. Extracts from hearts and airway tissues (AW) of $Plk1^{-lox}$ and $Plk1^{smko}$ mice were immunoblotted for Plk1 and GAPDH. Plk1 expression in hearts is similar between $Plk1^{-lox}$ mice and $Plk1^{smko}$ mice. However, Plk1 expression in airway tissues is reduced in $Plk1^{smko}$ mice. Blots are representative of four identical experiments. **(B) House dust mite extract (HDME) exposure increases airway resistance (R_{aw}) in mice.** $Plk1^{-lox}$ mice were exposed to HDME or PBS for six weeks. Airway resistance in these mice was evaluated by using the FlexiVent system as previously described (Li J et al. JBC, 291: 23693-23703, 2016) (*$P < 0.05$, n = 10-11).

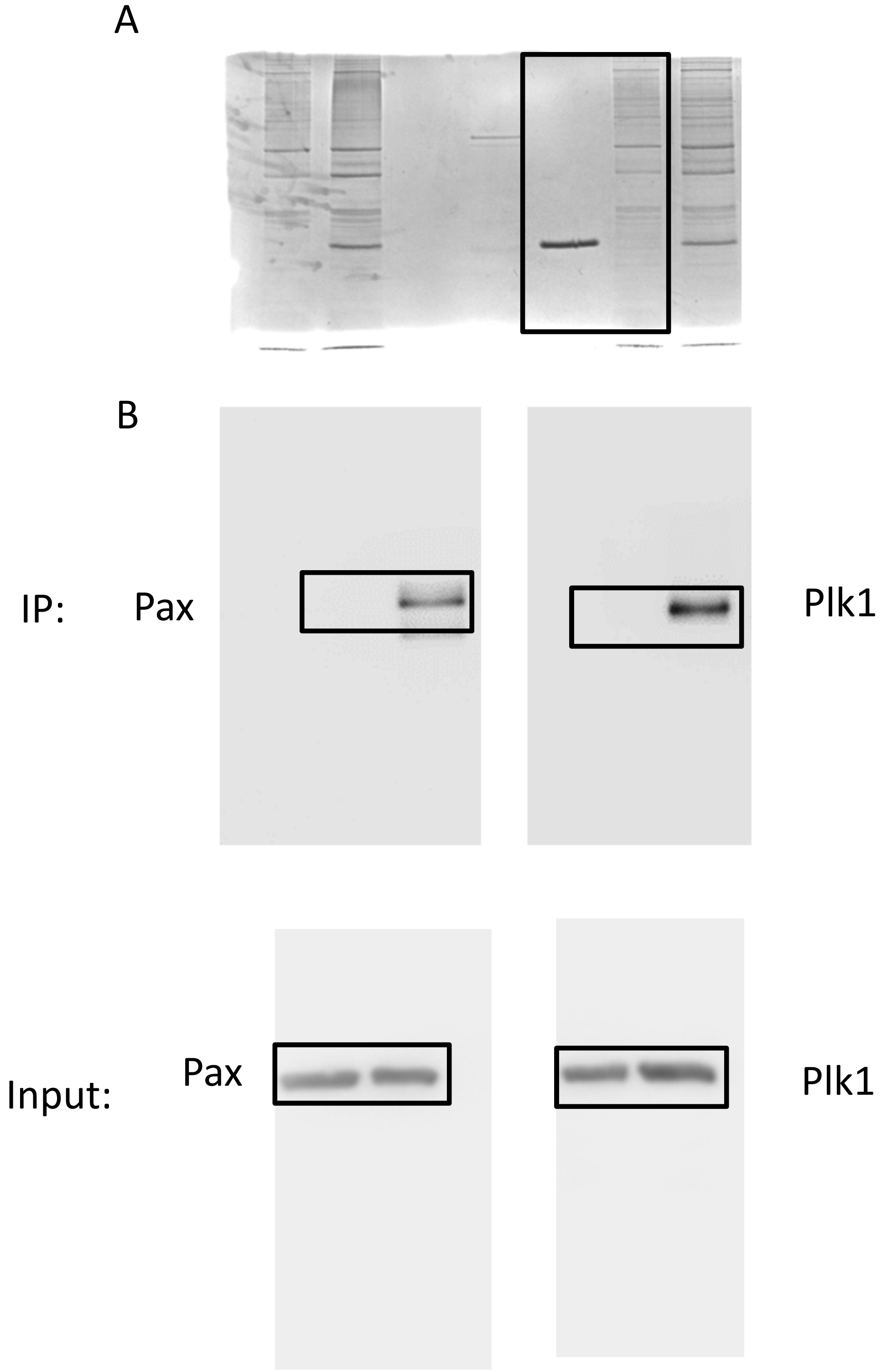

Figure S4. Original gels/blots for Figure 1A and B.

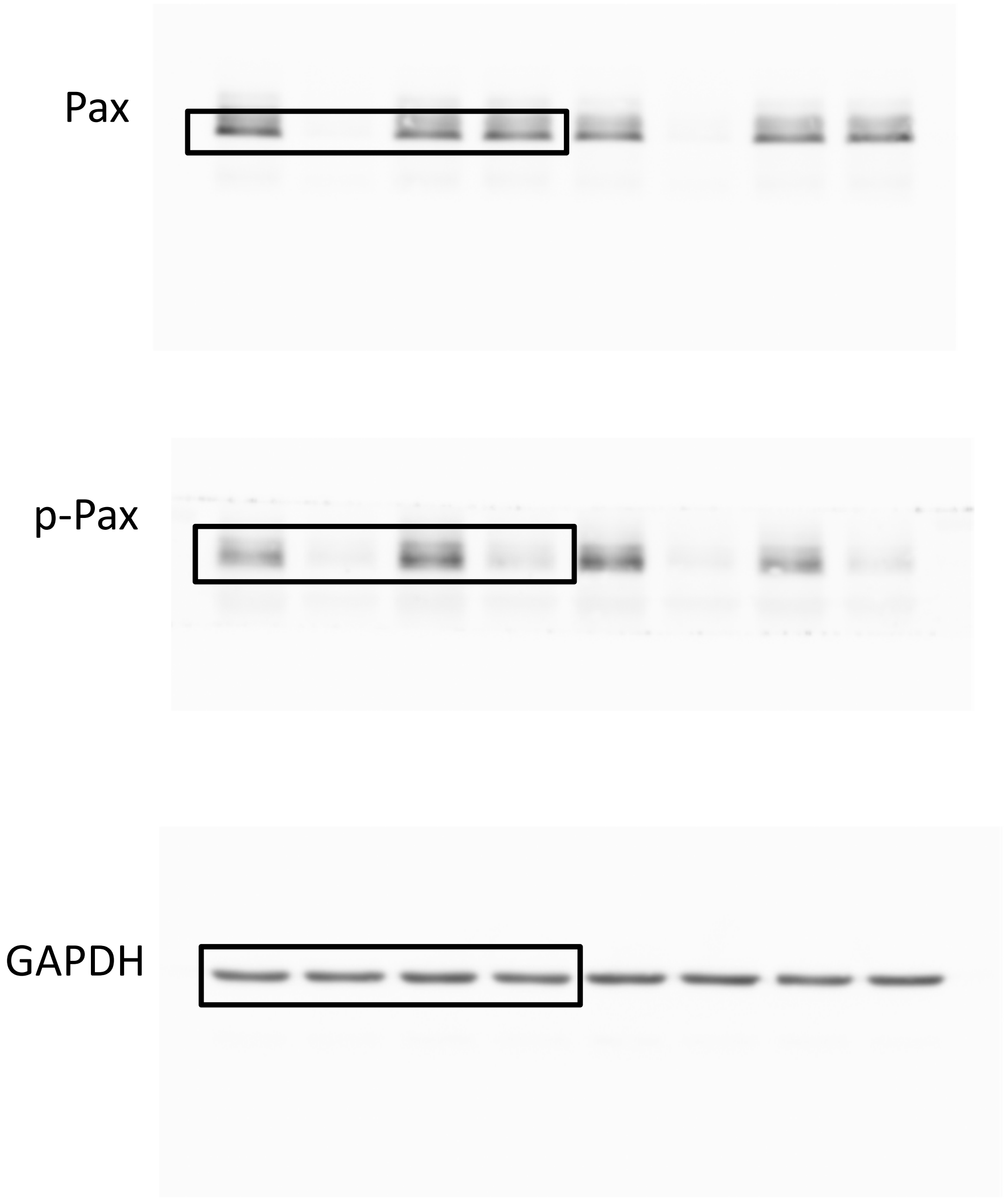

Figure S5. Original blots for Figure 1C.

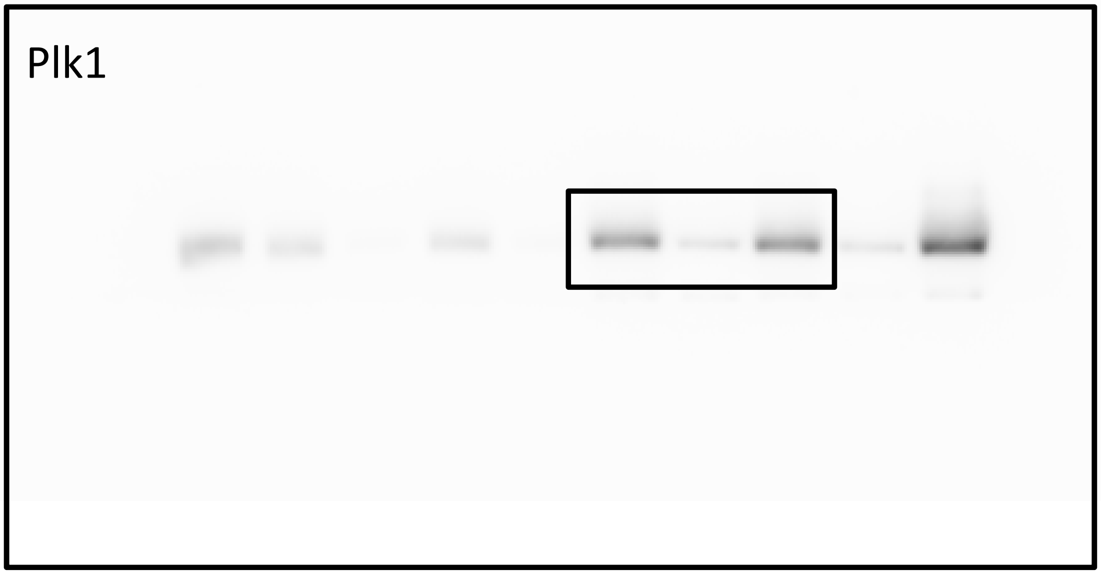

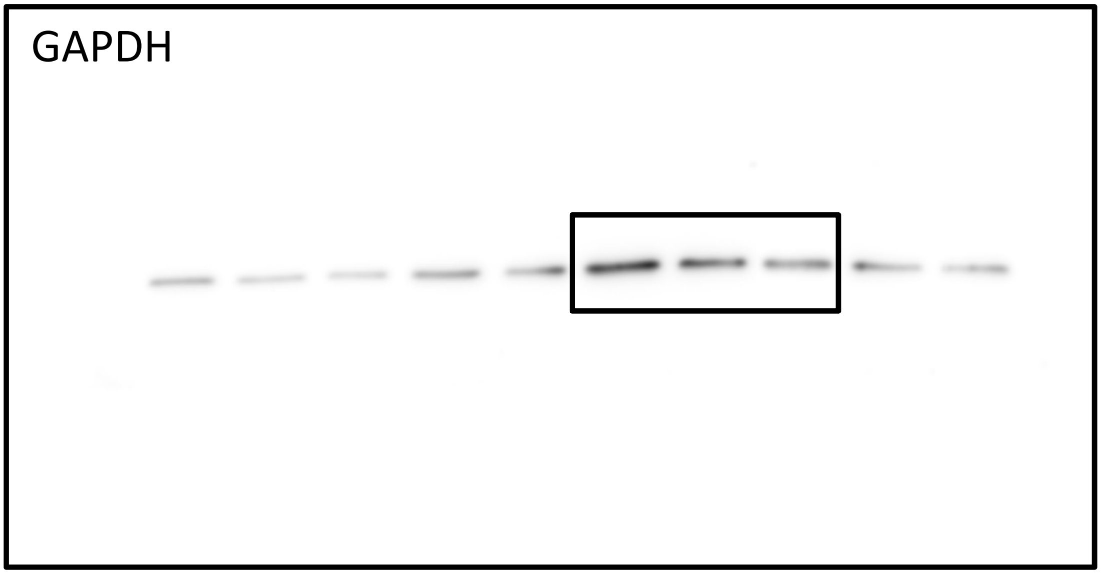

Figure S6. Original blots for Figure 4A.

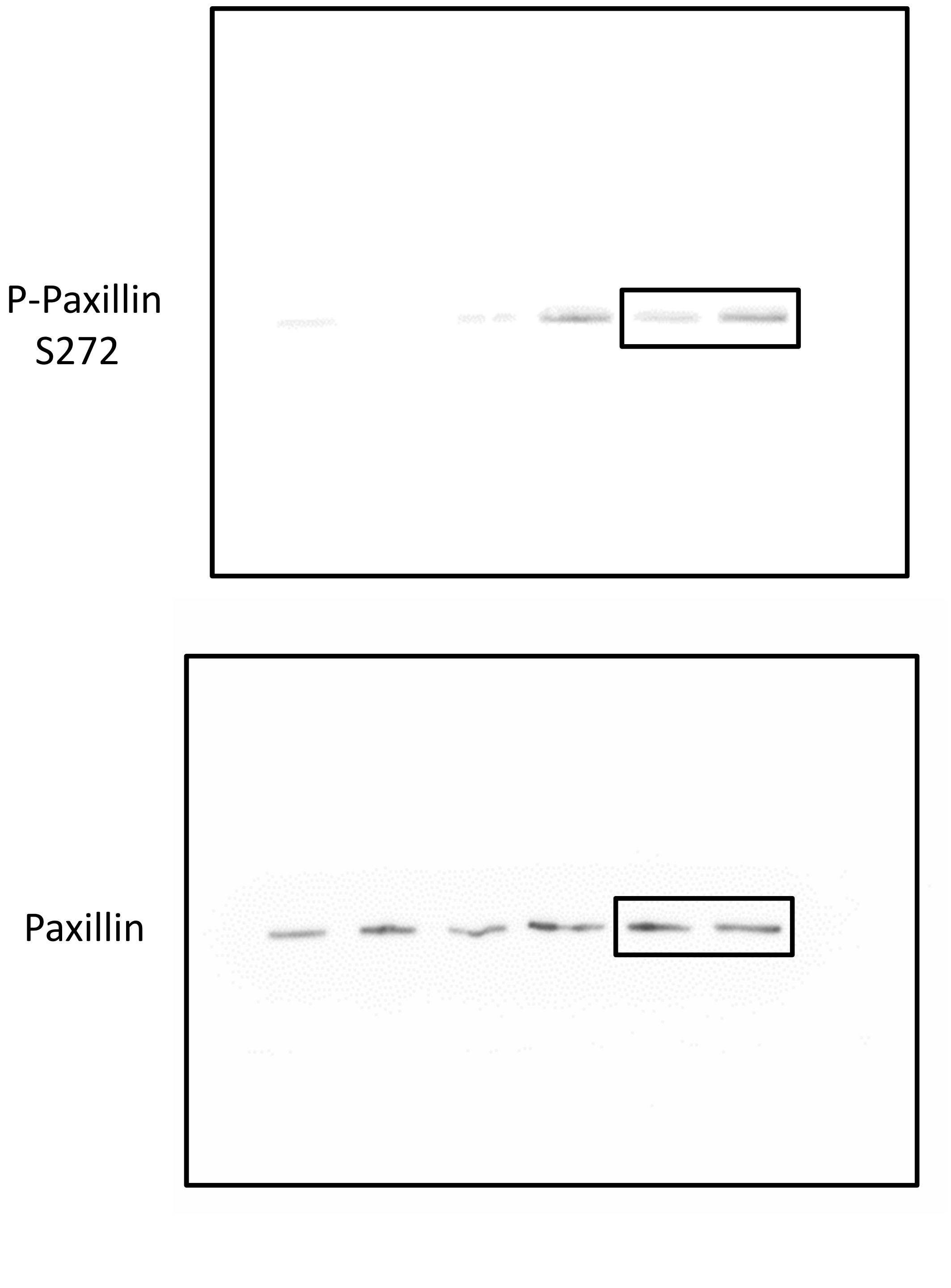

Figure S7. Original blots for Figure 4D.

P-Paxillin
S272

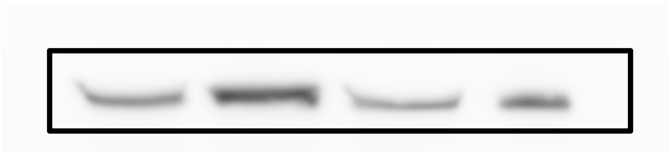

Paxillin

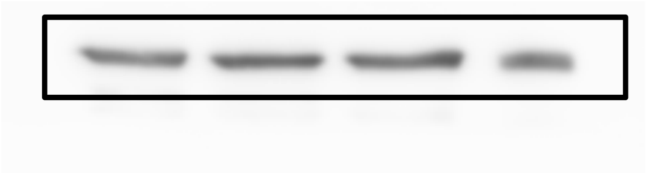

Figure S8. Original blots for Figure 5E.
